# EXPLORING THE ACTUAL SITUATION OF FAMILY CAREGIVING IN GER-DISTRICT IN THE SUBURBS OF ULAANBAATAR, MONGOLIA

**DOI:** 10.1093/geroni/igae098.2511

**Published:** 2024-12-31

**Authors:** Yumi Hashizume

**Affiliations:** Institute of Medicine, University of Tsukuba, Tsukuba, Ibaraki, Japan

## Abstract

Supporting family caregivers is going to be an increasing concern globally including developing countries. Mongolia, a developing country has challenges in caregiving due to lack of formal services and infrastructure development that family caregiving relies on mutual assistance by close relatives. This study aimed to explore the actual situation of family caregiving in District A in the suburbs of Ulaanbaatar. A questionnaire included caregiver education, nomadic living experience, financial status, support from relatives, CAGE (alcohol dependence), GDS5, coping behaviors, and JZBI8 (caregiving burden) distributed to 562 family-caregivers selected by district personnel. Of the 537 collected, 75 individuals involved in caregiving were divided, and statistically compared in to the three groups named as economically stable (ES: n1=29), partially in need (PIN: n2=35) and totally in need (TIN: n3=11). Mean values were below the cutoff point in all groups, and comparisons showed no differences except JZBI8. ES’ JZBI8 was significantly lower than PIN’s if the relative assisted purchasing medications and watching over care-recipients [F(2, 66）=3.93, p=.024, η2=.11]. ES with a higher education had more problem-focused-coping behaviors than TIN[F(2, 30）=3.64, p=.038, η2=.02]; however, ES did not receive laundry assistance if the relative had nomadic life. PIN engaged all-day caregiving had more avoidance-escape-coping-behaviors than non-all-day [t(27）=2.22, p=.035, d=0.92, 95%CI[0.07,1.77]]. PIN with nomadic experiences could not provide financial support to care-recipients [χ2(1，n1=31)=5.490, p=0.019, φ=0.421]. Although the burden of caregiving was generally low, family caregivers’ mutual support and coping behaviors vary by economic, educational, and nomadic background, which may develope into a caregiving burden for Mongolians.

